# Carriership of the rs113883650/rs2287120 haplotype of the *SLC7A5* (*LAT1*) gene increases the risk of obesity in infants with phenylketonuria

**DOI:** 10.1016/j.ymgmr.2020.100640

**Published:** 2020-08-21

**Authors:** Miroslaw Bik-Multanowski, Anna Madetko-Talowska, Iwona Betka, Elzbieta Swieczka, Bozena Didycz, Karolina Orchel-Szastak, Kinga Bik-Multanowska, Ewa Starostecka, Joanna Jaglowska, Renata Mozrzymas, Joanna Zolkowska, Katarzyna Chyz, Dorota Korycinska-Chaaban

**Affiliations:** a Department of Medical Genetics, Jagiellonian University Medical College, Krakow, Poland; b Department of Endocrinology and Metabolic Diseases, Polish Mother's Health Memorial Institute, Lodz, Poland; c Department Pediatrics, Hematology and Oncology, Medical University, Gdansk, Poland; d Voivodeship Hospital, Wroclaw, Poland; e Department of Inborn Errors of Metabolism and Paediatrics, Institute of Mother and Child, Warsaw, Poland

**Keywords:** Personalized medicine, Pharmacogenomics, Obesity, Metabolism, WES

## Abstract

**Purpose:**

Phenylketonuria (PKU) can be effectively treated with the use of a low-phenylalanine diet. However, some patients become overweight despite proper dietary treatment. We hypothesized that this phenomenon could be explained by the presence of specific variants within the genes involved in phenylalanine transport or in the phenylalanine transamination/oxygenation pathway.

**Methods:**

We selected a clinically homogenous group of 100 infants with PKU and assessed their growth patterns in the context of dietary phenylalanine tolerance. Next, within the sample, we performed exome sequencing and assessed a potential relationship between the observed phenotypical variability and the presence of structural variants in a priori selected genes of interest.

**Results:**

We detected a highly significant association between overweight and carriership of the rs113883650/rs2287120 haplotype of the *SLC7A5* (*LAT1*) gene, which encodes the main transmembrane transporter of large neutral amino acids and of thyroid hormones.

**Conclusions:**

Our findings suggest a pharmacogenetic effect of the relatively common rs113883650/rs2287120 haplotype of the *SLC7A5* gene. This can have practical implications for patients with PKU, since treatment protocols need to be reassessed to better prevent overweight in the carriers of the above variant.

## 1 Introduction

Phenylketonuria (PKU; MIM #261600) is a classic example of a monogenic disease with a genetic background that has been extensively studied [[1], [2], [3]]. Hyperphenylalaninemia, resulting from deficient activity of the enzyme phenylalanine hydroxylase (PAH) in the liver, is the major biochemical sign of PKU. The disease can be effectively treated with a low-phenylalanine diet [4]. However, the prediction of the metabolic phenotype based on individual genotype is not straightforward, even in patients with a complete absence of the PAH activity. Also, the wide genotypic diversity in most populations hinders the genotype-phenotype predictions [2,3]. Moreover, although the principles of treatment are well known, a proportion of patients who adhere well to dietary prescriptions tend to become overweight [[5], [6], [7], [8], [9], [10]]. Furthermore, blood phenylalanine concentrations do not always respond to the fluctuations of the phenylalanine intake [11,12]. These clinical observations suggest that the inconsistencies in the PKU phenotype could have a genetic background. This can relate to the transport of phenylalanine between various body compartments or to the additional PAH-independent pathways of phenylalanine metabolism that might become clinically significant in patients with severe hyperphenylalaninemia.

The transport mechanisms of phenylalanine through the various body pools, and its urinary excretion, remain not fully understood [[13], [14], [15], [16]]. However, five genes encoding the amino acid transporters are known to regulate the transmembrane transport of phenylalanine. Namely, the *SLC7A5* gene encoding the specific subunit of the LAT1 (L-type amino acid transporter 1), which exhibits the highest affinity to the phenylalanine, the *SLC7A8* gene (encoding the LAT2 transporter), the *SLC16A10* gene encoding the TAT1 (T-type amino acid transporter 1), as well as the *SLC43A1,* and the *SLC43A2* genes encoding the LAT3 and the LAT4 transporters, respectively.

The PAH-independent blood phenylalanine clearance is of low effectiveness [17]. However, the transamination of phenylalanine to phenylpyruvate and its further oxygenation should be considered in case of significant hyperphenylalaninemia. According to the Kyoto Encyclopedia of Genes and Genomes, five genes seem to be important in this regard: the *IL4I1* gene encoding the L-amino acid oxidase, the *GOT1* and the *GOT2* genes encoding the aspartate aminotransferase, the *TAT* gene encoding the tyrosine aminotransferase, and the *HPD* gene encoding the 4-hydroxyphenylpyruvate dioxygenase [18].

Currently, more than 950 pathogenic variants of the *PAH* gene have been described that result in the development of PKU, and most populations are genetically very heterogeneous [2,3]. Fortunately, the Polish population differs with this regard in comparison to the other countries, since approximately 40% of patients are homozygous for the variant p.Arg408Trp (commonly used name: R408W), which determines the nearly complete absence of the enzymatic activity of PAH [19].

In the present study, we aimed to assess the potential genetic background of the frequent overweight in infants with PKU, in the context of the variability of tolerance of dietary phenylalanine. We hypothesized that the previously mentioned phenotypic discrepancies between the patients could be explained by the presence of specific variants within the genes involved in the transmembrane transport of phenylalanine or the phenylalanine transamination/oxygenation pathway. We hoped that the knowledge gained in our study could contribute to the increased precision of dietary treatment strategies, and subsequently, could result in better prevention of the overweight in PKU.

## 2 Patients and methods

The study was conducted at the Department of Medical Genetics, Jagiellonian University Medical College in Krakow, Poland. The patients were recruited from five regional centers of the metabolic pediatrics in Poland (Krakow, Warsaw, Lodz, Gdansk and Wroclaw), which follow approximately 2500 patients with PKU (roughly 60% of the PKU population in Poland). In the present study, we only included the patients homozygous for the p.Arg408Trp variant (genotype: ENST00000553106.5: c.[1222C > T];[1222C > T]), who were born between the years 2000 and 2018. Estimation of sample size revealed that statistically significant results could be obtained if at least 86 individuals are recruited for the study (1-alpha = 95%; 1-beta = 80%; ratio of carriers of a gene variant/wild-type individuals = 1; percent of unexposed with outcome vs. exposed with outcome 5:30). Given the potential necessity for using correction for multiple comparisons, we finally recruited one hundred consecutive patients/infants; 47 girls and 53 boys.

First, we measured the body weight at birth, after six months of life (± 3 weeks) and at the age of 1 year (± 3 weeks). We also measured the body length and calculated the body mass index (BMI) after 6 months of life and at the age of 1 year. For an assessment of the growth parameters, we utilized the *Z*-score (Standard Deviation score) and the growth charts recommended for the Polish population [20]. Measurements of body weight and of body length were conducted by hospital anthropologists or metabolic nurses (body length was measured with the use of an infant measuring board).

Next, the dietary phenylalanine tolerance was estimated for all the patients by the dieticians taking care of them on an every-day basis. All patients were treated with the use of standard dietary protocol: recommended blood phenylalanine concentration of 0.12–0.36 mmol/L, a daily protein intake of approx. 2.4 g/kg of body weight. Blood phenylalanine concentration was controlled on a weekly basis (blood samples were collected at home by the parents of a child or at the outpatient clinic on the occasion of a control visit). During the observation, the mean blood phenylalanine concentrations remained within the recommended range in all patients. Incidental episodes of too high or too low concentrations were closely monitored and promptly corrected by dieticians taking care of the children.

For every patient, we calculated daily phenylalanine tolerance at the timepoints of the body weight assessment. We also assessed mean energy intake, total protein intake, and feeding mode: breast milk or infant formula as the source of natural protein. Only breast milk or commercially available standard infant formulas constituted the sources of natural protein. We assumed that 100 mL of breast milk contains 46 mg of phenylalanine. In breastfed infants, the individually calculated, appropriate amount of breast milk was mixed with phenylalanine-free formula prior to feeding. We excluded all preterm babies and infants with an additional gastrointestinal problem (such as gastroesophageal reflux and feeding intolerance) from the analysis.

Lastly, we assessed the presence of the genetic variants of the selected ten genes: *IL4I1*, *GOT1*, *GOT2*, *TAT*, *HPD*, *SLC7A5, SLC7A8, SLC16A10, SLC43A1,* and *SLC43A2*. The DNA for genotyping was extracted from the blood leukocytes (the blood samples of 1 mL were collected from every patient during the routine control blood tests). The MagCore Nucleic Acid Extractor (TK Biotech, Warsaw, Poland) was utilized for the extraction. Subsequently, 100 ng of genomic DNA of every sample was enzymatically fragmented (as determined at Sure Select XT and XT Low Input Enzymatic Fragmentation Protocol, Agilent Technologies, Santa Clara, USA) and used for exome library preparation according to the SureSelectXT HS Target Enrichment System for Illumina Paired-End Multiplexed Sequencing Library protocol (Agilent Technologies). Next, the libraries were tagged, pooled, and prepared for sequencing. Finally, the sequencing of the libraries was performed by an external genotyping service provider (Macrogen Europe, Netherlands) with the use of the NovaSeq 6000 genomic sequencer. The genotyping findings were confirmed with the use of classic sequencing (the Sanger's method).

The raw genotyping data were processed with the use of the DRAGEN Enrichment software (Illumina, San Diego, USA) in order to obtain a table of all genetic variants in every patient. However, the final data analysis was restricted only to the a priori selected set of ten genes encoding the phenylalanine transmembrane transporters and the enzymes of the phenylalanine transamination/oxygenation pathway. We also excluded rare genetic variants from the statistical analysis and focused only on those variants revealing the allelic frequency above 1%.

Finally, within the sample, we evaluated the presence of potential associations of the specific gene variants with the body weight and the phenylalanine tolerance. For the statistical analysis, we used descriptive statistics, the Pearson correlation and the *t*-test with a Bonferroni correction for multiple comparisons.

The study procedure was approved by the Jagiellonian University Ethics Committee (approval No. 1072.6120.9.2019). Informed consent form was obtained from the parents of the study participants.

## 3 Results

We assessed a group of 100 patients (47 girls and 53 boys). There were no apparent differences in the body weight in comparison to the general population of term babies at birth. The mean body weight equaled 3348 g (−0.15 SD) in girls and 3542 g (+0.12 SD) in boys. However, the mean body weight increased visibly for the entire group, reaching 7984 g (+0.59 SD) in girls and 8616 g (+0.49 SD) in boys at the age of six months. At the age of 1 year, the mean body weight equaled 10,456 g (+0.8 SD) in girls and 10,649 g (+0.26 SD) in boys. The mean Body Mass Index (BMI) value reached 17.84 (+0.89 SD) in girls at 6 months and 18.32 (+0.88 SD) at 1 year, whereas in boys the values were 18.36 (+0.89 SD) and 18.38 (+0.71 SD) after 6 months of life and 1 year, respectively. This suggests the presence of a tendency to develop overweight in some infants with PKU, especially during the first 6 months of life.

In our patients, the mean daily phenylalanine tolerance at the time point of the introduction of the therapeutic low-phenylalanine diet (first month of life) reached 138 mg (85–211 mg; 23–58 mg/kg). Subsequently, it increased to 275 mg (188–432 mg; 22–48 mg/kg) at the age of 6 months and remained nearly unchanged in the next 6 months, reaching 278 mg (214–389 mg; 19–36 mg/kg, mean 26.3 mg/kg) at the age of 1 year. Pearson correlation revealed that the total daily tolerance in the assessed infants positively correlated with their body weight in the first month of life: *r* = 0.42, *p* = 0.000 (significant at 0.01 level, 2-tailed), as well as in the sixth month of life: *r* = 0.44, p = 0.000 (significant at 0.01 level, 2-tailed).

The assessment of the feeding type revealed that breastmilk was a source of natural protein in 29% infants, and formula in the remaining 71% of patients. We did not observe any significant associations of the body growth dynamics or the phenylalanine tolerance neither with the type of feeding in study participants, nor with the phenylalanine tolerance.

Based on the above findings, in the final data analysis, we focused on two most interesting variables: body weight in the sixth month of life, when the apparent increase in average body weight was first observed, and dietary phenylalanine tolerance (mg/kg of body weight) at treatment initiation, reflecting the biggest observed interindividual differences in patients. We further analyzed potential associations of these two variables with the detected gene variants.

The sequencing of coding regions of the ten targeted genes identified 34 variants with an allelic frequency above 1%, which were further statistically evaluated. Among the variants of the *SLC7A5* gene, we identified two variants that are in complete linkage disequilibrium: rs113883650 and rs2287120 [21].

Among the analyzed 34 variants, an independent *t*-test revealed that there was a highly significant difference between the standardized (SD score) body weight of the carriers of the rs113883650/rs2287120 haplotype (1.12 ± 1.17) and individuals with the wild-type genotype of the *SLC7A5* gene (0.07 ± 1.20) at the age of six months (t(98) = −4.33, *p* = 0.00003), with the difference of −1.045 (95% CI, −1.522 to −0.568). The Bonferroni correction for multiple comparisons yielded a *p*-value of 0.002. The carriers of the above haplotype at the age of six months were, on average, much heavier than the wild-type individuals. Details on the detected gene variants can be found in Table 1.

**Table 1:** Gene variants with allelic frequency > 1% that were detected during the study and their association with body weight and dietary phenylalanine tolerance.

Gene ID	Gene variant (dbSNP ID)	Sequence context / consequence	Reference / variant allele	Allelic frequency (minor allele)	Significance of differences (p-value) between wild-type individuals and carriers of the detected gene variants (heterozygotes + homozygotes)	
					Body weight in the 6th month of life (Standard Deviation score)	Phenylalanine tolerance at treatment initiation (mg/kg)
*IL4I1*	rs146512348	Intron variant	G / A	0.08	0.98	0.45
	rs3810269	Intron variant	G / C	0.04	0.41	0.24
	rs546014086	Intron variant	GC / G	0.48	0.43	0.62
	rs1290754	Coding, synonymous variant	T / G	0.49	0.49	0.56
	rs892028	Intron variant	G / A	0.06	0.63	0.57
	rs1062798	Intron variant	C / G	0.35	0.17	0.57
	rs999583	Intron variant	A / G	0.22	0.68	0.71
*GOT1*	rs2234971	Coding, synonymous variant	C / T	0.06	0.24	0.62
*GOT2*	rs30842	Coding; missense variant	C / A	0.3	0.56	0.41
	rs1058192	Coding, synonymous variant	A / G	0.35	0.95	0.25
	rs11076256	Coding; missense variant	C / T	0.12	0.67	0.73
	rs14221	Coding, synonymous variant	C / A	0.37	0.42	0.06
	rs257636	Coding, synonymous variant	G / A	0.37	0.68	0.11
	rs11558171	Coding; missense variant	C / A	0.07	0.46	0.97
*TAT*	rs78302875	Coding, synonymous variant	C / T	0.05	0.55	0.35
	rs74344827	Coding; missense variant	G / A	0.16	0.42	0.58
*HPD*	rs1795963	Intron variant	G / A	0.37	0.16	0.53
	rs2247291	Intron variant	C / T	0.12	0.17	0.97
	rs1154510	Coding; missense variant	C / T	0.13	0.88	0.78
	rs2707072	Intron variant	T / A	0.07	0.18	0.93
*SLC7A5*	rs33996472	5’ UTR variant	G / A	0.09	0.91	0.98
	rs17853938	Coding, synonymous variant	G / T	0.15	0.94	0.58
	rs33975475	Intron variant	G / A	0.05	0.94	0.95
	rs113883650	Intron variant	TATATG / T	0.22	0.00003 (corrected *p* = 0.002)	0.85
	rs2287120	Intron variant	G / A	0.22	0.00003 (corrected p = 0.002)	0.64
*SLC7A8*	rs7157021	Coding, synonymous variant	A / G	0.45	0.69	0.43
	rs17183863	Coding, synonymous variant	G / A	0.07	0.71	0.85
	rs55816003	Intron variant	A / G	0.08	0.22	0.71
	rs1884545	Coding, synonymous variant	G / A	0.12	0.63	0.78
*SLC16A10*	rs33965856	Coding, synonymous variant	C / T	0.10	0.34	0.2
*SLC43A1*	rs112450479	Coding, synonymous variant	C / T	0.04	0.91	0.43
*SLC43A2*	rs72820319	Intron variant	G / A	0.06	0.10	0.007 (corrected *p* = 0.47)
	rs4790208	Intron variant	G / C	0.04	0.8	0.08
	rs12953268	Coding, synonymous variant	A / G	0.43	0.07	0.27

**Fig. 1:**
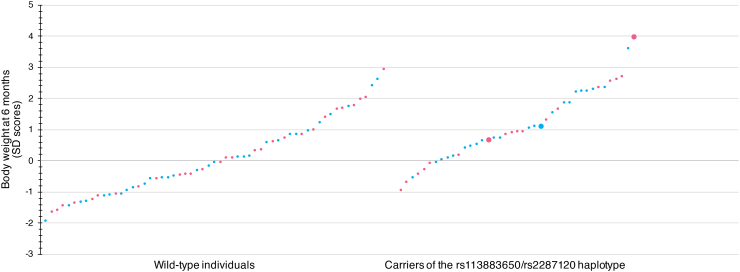
Differences in standard deviation scores for the body weight between wild-type individuals and carriers of the rs113883650/rs2287120 haplotype at the age of 6 months. The wild-type individuals are displayed on the left, and the carriers on the right. The carriers were, on average, much heavier. The difference in standardized body weight (SD score) was highly significant, as indicated by the *p*-value of 0.002 obtained by utilizing the Bonferroni correction for multiple comparisons. The pink and blue dots represent the girls and boys, respectively. Homozygotes are indicated by larger dots.

**Fig. 2:**
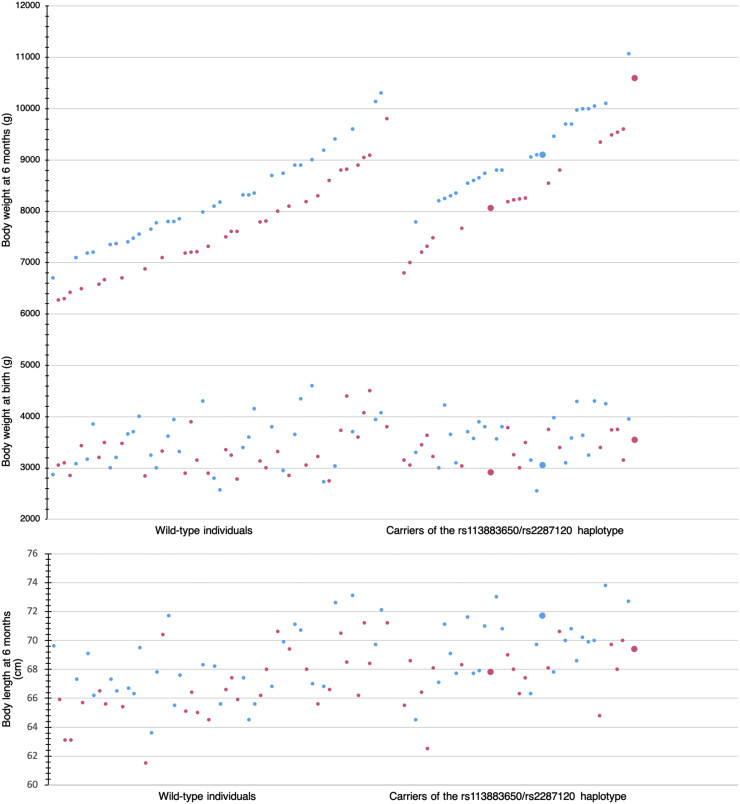
Body weight in grams at birth and at the age of 6 months, as well as body length at the age of 6 months in wild-type individuals and in carriers of the rs113883650/rs2287120 haplotype. The wild-type individuals are displayed on the left, and the carriers on the right. As can be seen, there were no visible differences in body weight at birth. However, differences in body weight became apparent at the age of 6 months (upper and middle parts of the figure), contrary to body length (lower part of the figure). The pink and blue dots represent the girls and boys, respectively. Homozygotes are indicated by larger dots.

Supplementary Body Mass Index analyses confirmed a clear tendency to become overweight in carriers. At the age of six 6, the average BMI in girls reached 19.1 in carriers vs. 17.39 in wild-type individuals (+1.84 SD vs. +0.56 SD), whereas in boys, the average BMI values were 19.39 vs. 18.00 (+1.7 SD vs. +0.62 SD), respectively. This tendency persisted at the age of 1 year. A detailed assessment of body weight and BMI with regards to the gender of study participants and to carriership of the rs113883650/rs2287120 haplotype can be found in Table 2, Fig. 1, and Fig. 2.

**Table 2:** Growth parameters in subgroups of infants with PKU participating in the study.

The studied population				Gender-specific reference data (general population)
		Carriers of the *SLC7A5* haplotype rs113883650/rs228712 (38 heterozygotes and 3 homozygotes)	No carriership of the *SLC7A5* haplotype rs113883650/rs228712 (59 cases)	
Girls	Body weight at birth	2910 g – 3780 g (mean 3382 g)	2750 g – 4500 g (mean 3325 g)	Mean 3350 g
	Body weight at six months	6800 g – 10,590 g (mean 8471 g)	6270 g – 9800 g (mean 7655 g)	Mean 7530 g (1 SD = 770 g)
	Body weight at one year	8420 g – 14,200 g (mean 10,926 g)	8200 g – 12,900 g (mean 10,129 g)	Mean 9540 g (1 SD = 1150 g)
	BMI at six months	19.1 (+1.84 SD)	17.39 (+0.56 SD)	Mean 16.65 (1 SD = 1.33)
	BMI at one year	18.72 (+1.1 SD)	17.98 (+0.69 SD)	Mean 16.76 (1 SD = 1.78)
Boys	Body weight at birth	3000 g – 4300 g (mean 3594 g)	2730 g – 4600 g (mean 3510 g)	Mean 3500 g
	Body weight at six months	7790 g – 10,100 g (mean 9145 g)	6700 g – 10,300 g (mean 8211 g)	Mean 8230 g (1 SD = 790 g)
	Body weight at one year	9850 g – 13,000 g (mean 11,459 g)	8700 g – 12,200 g (mean 10,086 g)	Mean 10,340 g (1 SD = 1210 g)
	BMI at six months	19.39 (+1.7 SD)	18.00 (+0.62 SD)	Mean 17.2 (1 SD = 1.29)
	BMI at one year	18.97 (+1.11 SD)	17.55 (+0.14 SD)	Mean 17.34 (1 SD = 1.47)

Assessment of the potential associations of the differences in the phenylalanine tolerance with the presence of the detected gene variants did not reveal any statistically significant findings. Also, the carriers of the rs113883650/rs2287120 haplotype did not differ significantly from wild-type individuals with regard to their mean daily intake of energy and of total protein, as estimated for the period from the end of the first month of life until the age of 6 months (116 kcal/kg versus 114 kcal/kg and 2.38 g/kg versus 2.39 g/kg, respectively). Data on individual patients can be found in Table 3.

**Table 3:** The mean individual daily intake of energy, protein and phenylalanine. The values were calculated for the period from the end of the first to the end of the sixth month of life.

Patient	Energy (kcal/kg)	Total protein (g/kg)	Phenylalanine (mg/kg)	Patient	Energy (kcal/kg)	Total protein (g/kg)	Phenylalanine (mg/kg)
Wild-type individuals							
Girls				Boys			
1	106	2.2	47	30	127	2.5	40
2	112	2.2	40	31	94	2.1	32
3	112	2.4	44	32	94	2.2	39
4	116	2.3	36	33	119	2.5	40
5	108	2.1	44	34	101	2.3	38
6	114	2.5	47	35	113	2.4	50
7	124	2.3	50	36	107	2.6	34
8	114	2.5	37	37	123	2.9	44
9	113	2.3	55	38	108	2.6	40
10	108	2.4	36	39	128	2.4	33
11	110	2.6	40	40	113	2.5	35
12	113	2.4	39	41	91	1.8	44
13	104	2.5	34	42	119	2.5	49
14	121	2.6	42	43	102	2.5	55
15	114	2.9	49	44	121	2.5	33
16	113	2.4	49	45	105	2.2	30
17	108	2.4	36	46	110	2	53
18	116	2.4	36	47	108	2.5	37
19	130	2.5	45	48	124	2.4	45
20	113	2.4	38	49	109	2.6	50
21	120	2.5	40	50	113	2.4	39
22	113	2.4	30	51	115	2.1	36
23	111	2.6	28	52	116	2.3	43
24	121	2.5	36	53	116	3	45
25	119	2.3	35	54	98	1.9	35
26	114	2.4	46	55	115	2.1	38
27	108	2.3	42	56	108	2.1	38
28	108	2.4	38	57	114	2.4	50
29	117	2.4	49	58	128	2.3	42
				59	106	2.3	45


Carriers of the rs113883650/rs2287120 haplotype							
Girls				Boys			
60	116	2.4	50	78	112	2.6	35
61	117	2.3	39	79	117	2.3	38
62	110	2.3	40	80	114	2.5	55
63	114	2.3	30	81	117	2.2	42
64	112	3	37	82	128	2.6	35
65	112	2.4	36	83	130	2.4	50
66	115	2.4	30	84	113	2.3	38
67	113	2.3	42	85	130	2.5	50
68	115	2.5	44	86	115	2.3	40
69	108	2.2	37	87	91	2.2	38
70	115	2.4	29	88	106	2.4	32
71	127	2.4	44	89	125	2.2	57
72	114	2.3	46	90	115	2.1	35
73	105	2.4	36	91	104	2.4	48
74	122	3	36	92	115	2.3	45
75	97	2.3	36	93	100	2.4	36
76	123	2.4	38	94	130	2.3	52
77	120	2.2	37	95	96	2.1	36
				96	120	2.5	40
				97	120	2.1	41
				98	119	2.3	52
				99	115	2.4	45
				100	114	2.6	34

## 4 Discussion

In the present study, we detected a clear tendency to develop overweight in patients with PKU, who are carriers of two coinherited variants of the *SLC7A5* gene: rs113883650 and rs2287120. The protein encoded by the *SLC7A5* gene – the L-type amino acid transporter 1 (LAT1) binds to the SLC3A2 to form a heterodimer. This heterodimer functions as the master transmembrane transporter of phenylalanine and other large neutral amino acids: tyrosine, leucine, histidine, methionine and tryptophan [[22], [23], [24], [25], [26]]. The transporter is expressed in most tissues, especially in the cerebral cortex, the blood-brain barrier, the bone marrow, the gastrointestinal tract, the placenta, and in several cancers [24,[27], [28], [29], [30]].

The rs113883650 variant is a deletion of five nucleotides (ATATG) located in intron 3 (ENSG00000103257; position 16: 87840502–87,840,508), within a close distance to the fourth exon. Interestingly, this genomic region seems to have a regulatory function with regard to gene expression as it encodes a binding site for the transcriptional repressor CTCF that regulates the 3D structure of chromatin. CTCF is thought to be a primary part of the activity of insulators, sequences that block the interaction between enhancers and promoter regions [31]. The rs2287120 variant is a single nucleotide polymorphism (G > A substitution) and is located in intron 6, near the start of exon 7 (ENSG00000103257; position 16:87837976). Both variants are located 2526 nucleotides from each other.

Our results strongly suggest the presence of a functional alteration in the *SLC7A5* gene in the carriers of the rs113883650/rs2287120 haplotype. However, it is not clear how the pathomechanism of becoming overweight could be related to the function of the gene.

Overweight and obesity represent key medical problems. Overweight can result from increased caloric intake, and in patients with inborn errors of metabolism, who are treated with the use of specific diets, the risk of overweight might increase due to inadequate metabolic control. However, experienced dietitians closely supervised the treatment in our patients and ensured that the diet was properly controlled. It should also be noted that all members of the metabolic teams used only one, common treatment protocol. Finally, the mean daily energy intake, as well as mean total protein and phenylalanine intakes, were nearly identical in carriers of the rs113883650/rs2287120 haplotype and in wild type individuals. Therefore, the dietary imbalance as a reason for becoming overweight seems to be unlikely in our patients. Similarly, the observed overweight can hardly be attributed to the alteration of the capacity of the SLC7A5/SLC3A2 complex for the transmembrane transport of neutral amino acids, which might potentially affect their incorporation into the body proteins and increase their conversion to the body fat.

However, the SLC7A5/SLC3A2 complex also mediates the transport of other biologically important substances: the thyroid hormones – triiodothyronine (T3) and thyroxine (T4), as well as the transport of several widely used and clinically important drugs (e.g., the antiparkinsonian drug L-Dopa, the anticancer drug melphalan and the antiepileptic drug gabapentin) [[32], [33], [34], [35], [36]]. Since the action of thyroid hormones is intracellular, alteration of their cellular uptake can alter the thyroid-dependent cellular metabolism and the basal metabolic rate, which might eventually lead to body weight increase. In this context, the presence of the rs113883650 variant that is localized in a regulatory, CTCF-binding region of the *SLC7A5* gene might have resulted in a functional alteration of the entire SLC7A5/SLC3A2 complex and in the observed tendency to overweight in our patients.

The carriership of the rs113883650/rs2287120 haplotype is common, and its potential association with overweight/obesity or with alteration of the homeostasis of thyroid hormones should have been detected in the previous genome-wide association studies in adult and pediatric populations [[37], [38], [39], [40]]. However, that did not happen. Nevertheless, additional factors make this situation more complex and should be considered. First, the cohort assessed in our study represents a group of young infants, who typically double their birth weight within the first 5 to 6 months of life. Even a subtle metabolic alteration in this period of very dynamic growth might significantly affect the weight gain. Moreover, feeding mode is similar in all young infants. Thus, the tendency to develop overweight in the carriers of the rs113883650/rs2287120 haplotype should be more clearly visible in this period of time in comparison to adults, who have their own individual dietary preferences and who can actively modify their body weight. Secondly, PKU is a rare disease that is genetically and clinically very heterogenic [2,3]. The previous studies on overweight in PKU included patients with various genotypes, disease severity, those following different dietary treatment regimens, and members of various age groups [6]. On the contrary, we selected a relatively large group of patients, who were homozygous for the most common pathogenic variant of *PAH* (p.Arg408Trp), belonged to one age group, and who could be precisely monitored with regard to their diet by dieticians. This approach allowed us to detect statistically significant genotype-phenotype correlations in relation to the a priori selected set of genes that could alter PAH-independent phenylalanine disposal in the body. Lastly, the rs113883650 variant might be of functional importance only in individuals with PKU, in whom blood phenylalanine concentrations are always well above the physiologic level. This is because hyperphenylalaninemia competitively blocks the SLC7A5/SLC3A2 complex-mediated transport of thyroid hormones [32]. Therefore, any subclinical alteration of cellular uptake of thyroid hormones in patients with PKU could be potentiated in carriers of the rs113883650 variant. On the contrary, hyperphenylalaninemia is not observed in persons not affected with PKU, and the functional effect of the rs113883650 variant should most probably be absent in them.

Future studies on cellular uptake of thyroid hormones in fibroblasts exposed to hyperphenylalaninemia could aim to verify the functional role of the rs113883650 variant of the *SLC7A5* gene.

We observed a fair association between phenylalanine tolerance and body weight. This correlation is expected and seems to reflect the body mass-related net protein catabolism-synthesis ratio. However, the observed large interindividual differences in phenylalanine tolerance suggest that additional factors might be responsible for this variability. Previous observations of the significant differences in the blood and urine concentrations of the phenylalanine transamination/oxygenation metabolites (phenylpyruvic acid, phenyllactic acid and hydroxyphenylacetic acid) in patients with similar blood phenylalanine concentrations seem to support this hypothesis [14]. However, it should be noted that the above-mentioned differences were observed mostly in patients with very high hyperphenylalaninemia, whereas in the participants within our study the blood phenylalanine concentration was carefully monitored. Therefore, further studies with patients with various severity of hyperphenylalaninemia are needed in order to explore the potential genetic background of the various tolerance of dietary phenylalanine.

Some limitations of the present study should be mentioned. We did not use any control population. Assessment of the growth dynamics of healthy infants could help to eliminate the bias related to potential dietary imbalance during the treatment of infants with PKU. Also, we did not assess patients from other countries, which could be useful to exclude any unexpected population-specific or region-specific genetic or epigenetic effects. The above issues can be addressed in further, confirmatory studies in larger populations.

## 5 Conclusions

Our study demonstrates a clinically relevant association between developing overweight in patients with PKU and the carriership of the common rs113883650/rs2287120 haplotype of the *SLC7A5* gene encoding the L-type amino acid transporter 1. Our findings might have potential practical implications for children with PKU, since treatment protocols need to be reassessed to better prevent overweight in the carriers of the above gene variant.

## Informed consent

Informed consent was obtained from the parents of patients participating in the study.

## Declaration of Competing Interest

The authors declare that they have no competing financial interests and no other conflicts of interest in connection with this paper.
